# LPAR1, Correlated With Immune Infiltrates, Is a Potential Prognostic Biomarker in Prostate Cancer

**DOI:** 10.3389/fonc.2020.00846

**Published:** 2020-06-10

**Authors:** Jingqi Shi, Dongbo Jiang, Shuya Yang, Xiyang Zhang, Jing Wang, Yang Liu, Yuanjie Sun, Yuchen Lu, Kun Yang

**Affiliations:** ^1^Department of Immunology, The Fourth Military Medical University, Xi'an, China; ^2^School of Basic Medicine, The Fourth Military Medical University, Xi'an, China

**Keywords:** lysophosphatidic acid receptor 1, tumor-infiltrating immune cells, chemokines, migration, prostate cancer

## Abstract

Prostate cancer is a common malignancy in men worldwide. Lysophosphatidic acid receptor 1 (LPAR1) is a critical gene and it mediates diverse biologic functions in tumor. However, the correlation between LPAR1 and prognosis in prostate cancer, as well as the potential mechanism, remains unclear. In the present study, LPAR1 expression analysis was based on The Cancer Genome Atlas (TCGA) and the Oncomine database. The correlation of LPAR1 on prognosis was also analyzed based on R studio. The association between LPAR1 and tumor-infiltrating immune cells were evaluated in the Tumor Immune Estimation Resource site, ssGSEA, and MCPcounter packages in R studio. Gene Set Enrichment Analysis and Gene Ontology analysis were used to analyze the function of LPAR1. TCGA datasets and the Oncomine database revealed that LPAR1 was significantly downregulated in prostate cancer. High LPAR1 expression was correlated with favorable overall survival. LPAR1 was involved in the activation, proliferation, differentiation, and migration of immune cells, and its expression was positively correlated with immune infiltrates, including CD4+ T cells, B cells, CD8+ T cells, neutrophils, macrophages, dendritic cells, and natural killer cells. Moreover, LPAR1 expression was positively correlated with those chemokine/chemokine receptors, indicating that LPAR1 may regulate the migration of immune cells. In summary, LPAR1 is a potential prognostic biomarker and plays an important part in immune infiltrates in prostate cancer.

## Introduction

Prostate cancer is a common malignancy in men worldwide (1). It is also the second leading cause of death related to cancer around the western countries. Although several drugs for patients who were suffering from castration-resistant prostate cancer have been approved (2, 3), such as enzalutamide and abiraterone, there is still an urgent need for treating patients who have no response to androgen depravation therapy. The tumor microenvironment (TME) has been reported to be associated with prostate cancer progression (4–6). Immunotherapy, a promising strategy, showed antitumor effects in prostate cancer (7, 8). Recent studies have found that the tumor-infiltrating immune cells affect the prognosis of a patient and the antitumor efficacy of immunotherapy (9–12). However, the molecular immune-related mechanisms in prostate cancer remain ambiguous. Therefore, the identification of novel therapeutic biomarkers associated with immune infiltrates in prostate cancer is urgently needed.

Lysophosphatidic acid receptor 1 (LPAR1) is one of the G protein-coupled receptors and binds with lysophosphatidic acid (LPA) (13). It is involved in diverse biological functions, including chemotaxis (14), proliferation (15), cell differentiation (16), platelet aggregation (17), and tumor progression (13). A set of papers indicated that LPAR1 is a prognostic biomarker in various cancers and takes an important part in the development of prostate cancer (18–20). However, the LPAR1-correlated functions and mechanisms in tumor immunology and tumor progression remain to be explored.

The rapid development of high-throughput sequencing makes it possible to explore the mechanisms in diseases (21). The Cancer Genome Atlas (TCGA) is a landmark project that contains 32 human cancers through genome sequencing, making an effort to understand the molecular basis of cancer. It has been made available in order to figure out the function of LPAR1 in prostate cancer at a large scale.

Integrative analysis and several visualization methods were used in this present study to explore the mechanism of LPAR1 in prostate cancer. We investigated the LPAR1 expression levels and analyzed the correlation of LPAR1 and the prognosis of patients. Gene Ontology (GO) analysis, Gene Set Enrichment Analysis (GSEA), and several methods were also utilized to explore the potential function of LPAR1 in tumor progression and immune microenvironment. The findings suggested the potential mechanisms of LPAR1, giving us new insights into the important role of LPAR1 in prostate cancer.

## Materials and Methods

### Data Source and Processing

The prostate adenocarcinoma (PRAD) clinical and molecular data (including mRNA expression and mutations) was extracted from the TCGA Data Portal (https://tcga-data.nci.nih.gov/tcga/) through the TCGAbiolinks (22) R package. In terms of the gene expression profile, we downloaded two types of data including raw counts data and transcripts per kilobase of per million mapped (TPM) data, one of the normalized gene expression estimations. We got the mRNA expression information of 52 normal patients and 499 tumor patients and the clinical information of 499 patients. In addition, the GSE6956 dataset was extracted from GEO database, including 69 tumor patients and 18 normal patients with prostate cancer.

### Oncomine Database Analysis

The Oncomine microarray database was used for analysis (https://www.oncomine.org/). We screened the mRNA levels of LPAR1 in various types of cancers. *P*-value <0.05 and fold change >2 were restricted as the thresholds.

### GO Analysis and GSEA

We separated all patients into two groups based on the median value of LPAR1 mRNA expression data. The log2 fold change and *p*-value calculated by DEseq2 (23) package were used as ranking metric. The GO terms (C5 collection in GSEA) were divided into three sub-collections: biological process (BP), molecular function, and cellular component. It is one of the most frequently used databases for pathway annotation. The two enrichment analyses were based on the BP sub-collection, which contains 7,350 genes. For the GO analysis, we used the Cytoscape (24) software and the ClueGO (25) app to analyze the function of differentially expressed genes (DEGs) with *p*-value < 0.01 and GO term network connectivity score equal to 0.6. As for GSEA, there is no need for the screening of differentially expressed genes. Hence, those genes that have a limit change in the transcriptional level but are functionally important can be retained. Compared to conventional GO and Kyoto Encyclopedia of Genes and Genomes enrichment analyses, GSEA retains more information. For GSEA, we made use of the clusterprofiler (26) R package in R studio, and the C5 collection was the gene set used in the present analysis.

### TIMER Database Analysis

The Tumor Immune Estimation Resource (TIMER) (27) is an integrative web server for evaluating tumor-infiltrating immune cells across diverse cancer types (https://cistrome.shinyapps.io/timer/). The TIMER includes more than 10,000 samples across multiple cancer types of the TCGA. It applies a partial deconvolution linear least square regression method to calculate the abundance of immune infiltrates. We evaluated the correlation between LPAR1 expression and immune infiltrates in tumors, including CD8+ T cells, CD4+ T cells, dendritic cells, neutrophils, B cells, and macrophages.

### Immune Infiltrates in Tumor Tissues

The Microenvironment Cell Populations counter (MCPcounter) method (28) and single-sample GSEA (ssGSEA) method (29) were used to calculate the level of tumor-infiltrating immune cells based on PRAD mRNA TPM data. The ssGSEA marker genes were extracted from the paper of Bindea et al. (30) and it included 24 types of immune cells. Figures were generated with a pheatmap R package.

### Protein–Protein Interaction Analysis

The LPAR1 and chemokines/chemokine receptors were searched in a protein–protein interaction (PPI) network *via* the STRING database (https://string-db.org/). The minimum required interaction cutoff is 0.4. The edges between nodes represent protein–protein associations. The edge with blue color means that the two nodes have known interactions from curated databases. The yellow color means textmining. The black color means that the two nodes have a co-expression. The purple color means that the interactions of the two nodes were experimentally determined.

### Statistical Analysis

The statistical analysis and the graphical work in this study were mainly conducted by R programming language with several packages, such as DEseq2 package, survival package, and TCGAbiolinks package. The survival curve based on log-rank test was depicted with Kaplan–Meier method. Univariate survival analysis was based on Cox proportional hazards model. Hazard ratio (HR) and log-rank test were used for comparing the overall survival between patients in different groups. Throughout the study, the threshold of statistical significance was *P* < 0.05.

## Results

### LPAR1 mRNA Expression Was Downregulated in Diverse Cancers

**Figure 1:**
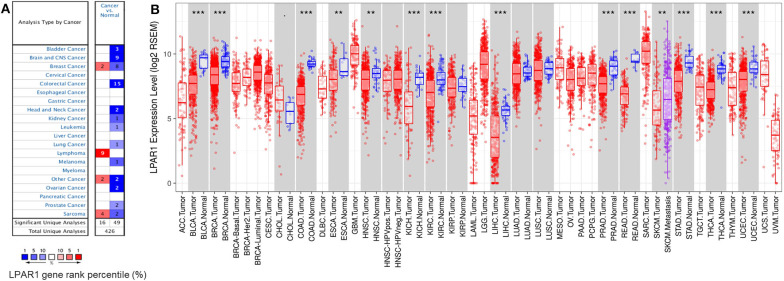
Lysophosphatidic acid receptor 1 (LPAR1) expression levels in different kinds of human cancers. **(A)** LPAR1 profile in different types of human cancers compared with normal tissues based on the Oncomine database. **(B)** The LPAR1 expression levels between tumor and normal tissue among all The Cancer Genome Atlas datasets were analyzed by the Tumor Immune Estimation Resource (**P < 0.01, ***P < 0.001).

To compare the mRNA expression levels of LPAR1 in normal and tumor tissues, we used the Oncomine database to determine the LPAR1 expression among multiple cancer types. This analysis indicated that LPAR1 was highly expressed in lymphoma and lowly expressed in prostate cancer, bladder cancer, brain and central nervous system cancer, head and neck cancer, colorectal cancer, kidney cancer, lung cancer, leukemia, melanoma, and ovarian cancer (Figure 1A). To further validate the LPAR1 expression in different cancers, we explored the differential gene expression between tumor and normal tissue among all TCGA datasets *via* TIMER database and show it in Figure 1B. LPAR1 was significantly lowly expressed in PRAD, breast invasive carcinoma (BRCA), bladder urothelial carcinoma (BLCA), colon adenocarcinoma (COAD), rectum adenocarcinoma (READ), head and neck cancer (HNSC), esophageal carcinoma (ESCA), kidney renal clear cell carcinoma (KIRC), kidney chromophobe (KICH), liver hepatocellular carcinoma (LIHC), thyroid carcinoma (THCA), stomach adenocarcinoma (STAD), and uterine corpus endometrial carcinoma (UCEC). In brief, LPAR1 was downregulated in colorectal cancer, breast cancer, kidney cancer, head and neck cancer, and prostate cancer based on the Oncomine database and TCGA.

### Association of LPAR1 Expression and Immune Cell Populations

**Figure 2:**
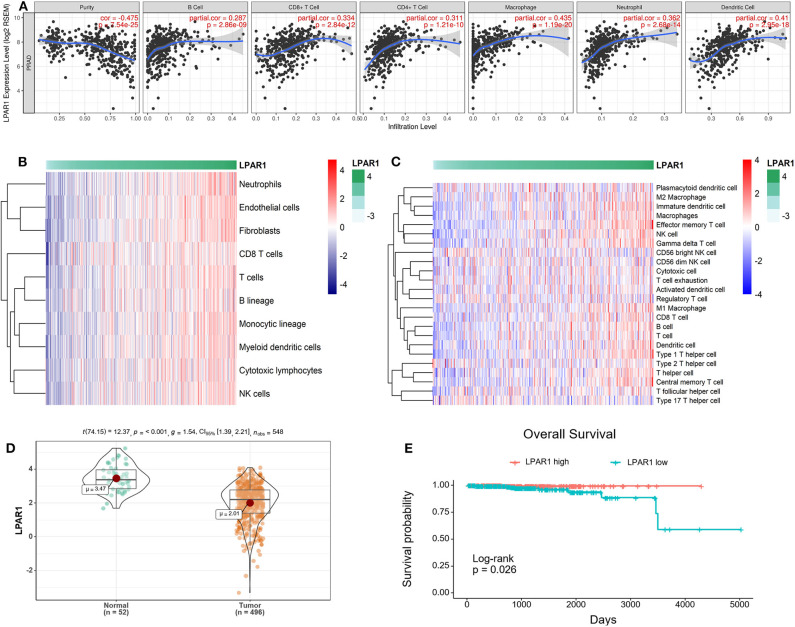
Correlation of lysophosphatidic acid receptor 1 (LPAR1) expression and tumor-infiltrating immune cells and prognosis in prostate adenocarcinoma (PRAD). **(A)** LPAR1 expression was significantly negatively related to tumor purity and had a positive correlation with the abundance of several immune cell types in PRAD, including CD8+ T cells, CD4+ T cells, macrophages, B cells, neutrophils, and dendritic cells. **(B)** Heatmap of the correlation between LPAR1 and T cells, B lineage, monocytic lineage, myeloid dendritic cells, cytotoxic lymphocytes, and natural killer cells as performed by the MCPcounter R package. Higher LPAR1 expression was associated with higher abundance of immune infiltrates in PRAD. **(C)** Heatmap of the correlation between LPAR1 and several immune cells based on ssGSEA R package. **(D)** Violin plot comparing the LPAR1 expression for patients with highly expressed LPAR1 *vs*. those with lowly expressed LPAR1 in prostate cancer with *p*-value < 0.001. **(E)** Kaplan–Meier plot comparing the overall survival for patients with highly expressed LPAR1 *vs*. those with lowly expressed LPAR1 in prostate cancer using log-rank test with *p*-value < 0.05.

The association between LPAR1 and tumor-infiltrating immune cells in multiple cancer types was based on the TIMER database, including breast cancer, head and neck cancer, colorectal cancer, kidney cancer, and prostate cancer (Figure S1). We found that LPAR1 impacted tumor-infiltrating immune cells in prostate cancer (Figure 2A). The LPAR1 expression was negatively correlated with the purity of tumor (*r* = −0.475, *P* = 7.54e−25), Furthermore, the LPAR1 expression was positively correlated with the abundance of several immune cell types, including CD4+ T cells (*r* = 0.311, *P* = 1.21e−10), CD8+ T cells (*r* = 0.334, *P* = 2.84e−12), neutrophils (*r* = 0.362, *P* = 2.68e−14), macrophages (*r* = 0.435, *P* = 1.19e−20), and dendritic cells (*r* = 0.41, *P* = 2.95e−18) in PRAD. To validate these findings, we used the MCPcounter method. We evaluated the association between LPAR1 and tumor-infiltrating immune cells from the mRNA expression data. A strong positive correlation between LPAR1 and myeloid dendritic cells, T cells, B lineage, monocytic lineage, cytotoxic lymphocytes, and natural killer (NK) cells was seen (Figure 2B). In addition, the ssGSEA analysis (Figure 2C) revealed that LPAR1 was positively correlated with the infiltration of γδ T cells, effective memory T cells, central memory T cells, type 1 T helper cells, CD8+ T cells, dendritic cells (DCs), M1 macrophages, and B cells and negatively correlated with CD56 bright NK cells and Treg cells. In addition, LPAR1 was also positively correlated with tumor-infiltrating immune cells based on the GSE6956 dataset (Figure S2). Hence, LPAR1 may play critical roles in regulating antitumor immunity.

### LPAR1 Expression Level Was Associated With the Prognosis of Patients With Prostate Cancer

The downregulation of LPAR1 was validated by using the TCGA-PRAD dataset (Figure 2D). To gain deeper insights into the tumor mechanisms in human prostate cancer, we performed analyses to reveal the relevance between LPAR1 and the prognosis of patients with prostate cancer. Interestingly, as analyzed by Kaplan–Meier plot and log-rank tests, LPAR1 was correlated with the patients' clinical outcome (Figure 2E). In addition, we did a univariate cox proportional hazards regression analysis and found that a high LPAR1 expression was correlated with a favorable overall survival (OS) (HR = 0.51, *P* = 0.00462) in prostate cancer, suggesting that LPAR1 expression can impact the prognosis of patients with prostate cancer.

### LPAR1 Was Related to Several Immune-Related Pathways in Prostate Cancer

**Figure 3:**
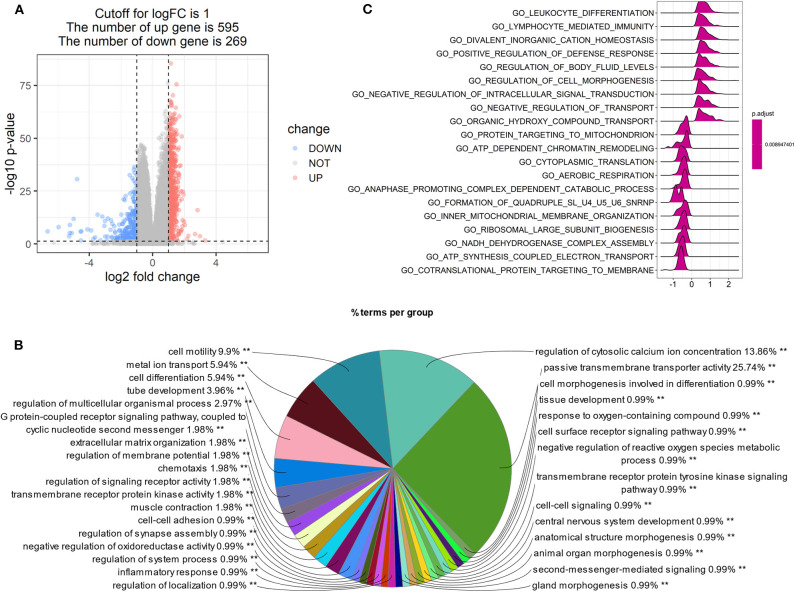
Gene Ontology (GO) and Gene Set Enrichment Analysis (GSEA) of tumor samples with high expression of lysophosphatidic acid receptor 1 (LPAR1) *vs*. those with low expression of LPAR1. The analysis was based on the biological process category in GO. **(A)** Volcano plot showing the differentially expressed genes (DEGs) comparing tumor samples between samples with high LPAR1 expression and samples with low LPAR1 expression. **(B)** The pie plot only showed the pathways with *p*-value < 0.01 and the GO term network connectivity score is 0.6. The upregulated DEGs were enriched in a set of pathways, including passive transmembrane transporter activity, cell motility, metal ion transport, cell differentiation, tube development, and G protein-coupled receptor signaling pathway. **(C)** The plot shows the top 20 categories with *p*-value < 0.05 *via* GSEA, including leukocyte differentiation, lymphocyte-mediated immunity, divalent inorganic cation homeostasis, positive regulation of defense response, regulation of body fluid levels, regulation of cell morphogenesis, and so on.

Patients with high LPAR1 expression had a prolonged OS time, suggesting that LPAR1 may be involved in the initiation and the progression of prostate cancer. Then, we analyzed the RNA sequencing data downloaded from TCGA and compared the tumor samples between samples with high LPAR1 expression and samples with low LPAR1 expression. The volcano plot was shown in Figure 3A and DEGs were shown in Table S1, including 595 upregulated genes and 269 downregulated genes. We used upregulated genes in high LPAR1 patients for GO analysis. The GO analysis showed that the DEGs were enriched in a set of pathways, including passive transmembrane transporter activity, cell motility, metal ion transport, cell differentiation, tube development, G protein-coupled receptor signaling pathway, and chemotaxis (Figure 3B). In addition, the downregulated genes were enriched in pathways, including the detection of chemical stimulus, cell proliferation in the external granule layer, and telencephalon cell migration (Figure S3). The GSEA indicated the enrichment in many categories, such as leukocyte differentiation, lymphocyte-mediated immunity, divalent inorganic cation homeostasis, positive regulation of the defense response pathway, and regulation of cell morphogenesis (Figure 3C). We used the whole gene set after sorting for GSEA. The results of GSEA showed that LPAR1 participated in various functions and pathways, including antitumor immune responses.

**Figure 4:**
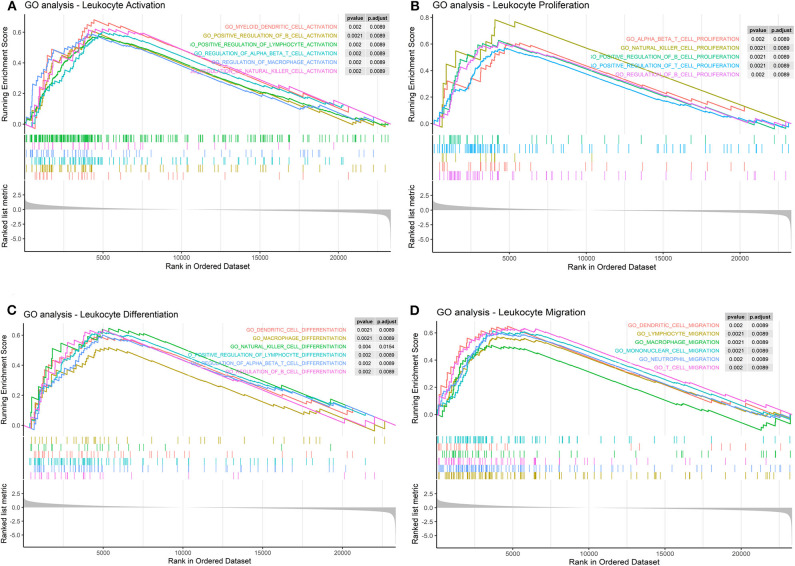
Immune cell-related pathways based on the Gene Set Enrichment Analysis (GSEA). **(A)** Lysophosphatidic acid receptor 1 (LPAR1) was associated with the activation of T cell, natural killer (NK) cell, B cell, dendritic cell (DC), and macrophage in Gene Ontology (GO) terms. **(B)** LPAR1 was associated with the proliferation of T cell, NK cell, and B cell in GO terms. **(C)** LPAR1 was associated with the differentiation of T cell, NK cell, B cell, DC, and macrophage in GO terms. **(D)** LPAR1 was associated with the migration of T cell, DC, neutrophil, and macrophage in GO terms.

Furthermore, we found that LPAR1 can take part in several immune-related pathways and influence many processes of immune cells. The results indicated that LPAR1 was associated with the activation (Figure 4A) of T cell, NK cell, B cell, DC, and macrophage, the proliferation (Figure 4B) of T cell, NK cell, and B cell, the differentiation (Figure 4C) of T cell, NK cell, B cell, DC, and macrophage, and the migration (Figure 4D) of T cell, DC, neutrophil, and macrophage.

### Linear Correlation and PPI Network Between LPAR1, Chemokines, and Chemokine Receptors

**Figure 5:**
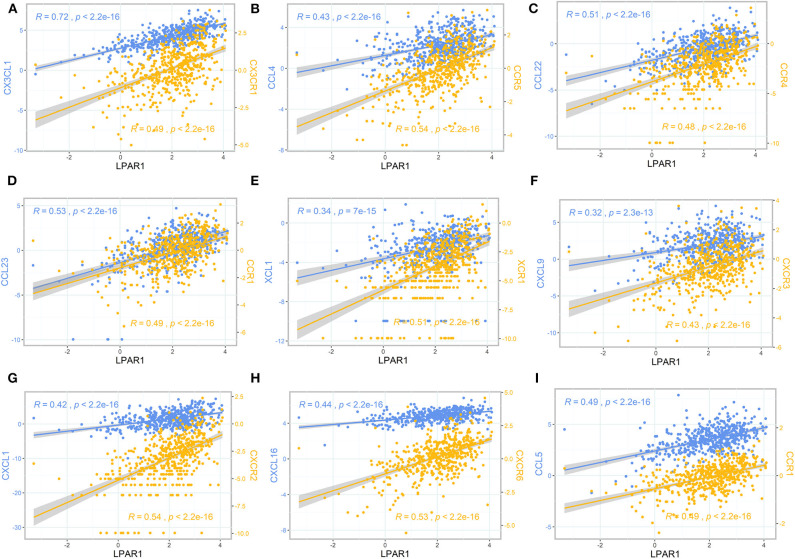
The scatter plot and correlation curve of lysophosphatidic acid receptor 1 (LPAR1), chemokines, and chemokine receptors. **(A–I)** LPAR1 was positively associated with CX3CL1, CX3CR1, CCL4, CCR5, CCL22, CCR4, CCL23, CCR1, XCL1, XCR1, CXCL9, CXCR3, CXCL1, CXCR2, CXCL16, CXCR6, CCL5, and CCR1. CCL, CC chemokine ligand; CCR, CC chemokine receptor; CXCR, CXC chemokine receptor; CXCL, CXC chemokine ligand; XCL, C chemokine ligand; XCR, C chemokine receptor; CX3CL, CX3C chemokine ligand; CX3CR, CX3C chemokine receptor.

To further clarify the association between LPAR1 and immune cell migration, we integrated chemokines and chemokine receptors in Figures 5A–I. As the figures show, LPAR1 expression was positively correlated with lymphocyte-associated chemokines and chemokine receptors, including CX3CL1, CX3CR1, CCL4, CCR5, CCL22, CCR4, CCL23, CCR1, XCL1, XCR1, CXCL9, CXCR3, CXCL1, CXCR2, CXCL16, CXCR6, CCL5, and CCR1. The correlation curves with two different y axes were performed by ggplot2 R package. Those chemokine and chemokine receptors that seemed to be upregulated with LPAR1 expression level increased. Hence, high LPAR1 expression may contribute to the migration of immune cells to the tumor tissues.

**Figure 6:**
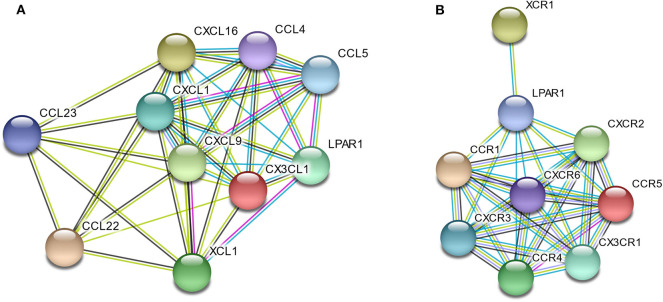
Protein–protein interactions (PPI) network based on lysophosphatidic acid receptor 1 (LPAR1) and chemokines/chemokine receptors with a minimum required interaction score > 0.40. **(A)** PPI network among LPAR1 and nine chemokines. **(B)** PPI network among LPAR1 and eight chemokine receptors.

To better understand the interactions among LPAR1 and chemokines or chemokine receptors, the STRING database was utilized to generate and visualize a PPI network. The PPI network showed that LPAR1 had known or predicted interactions with the various chemokines studied above (Figure 6A), including 10 nodes and 38 edges. The LPAR1/CCL5, LPAR1/CCL4, and LPAR1/XCL1 interactions were experimentally determined. The interactions of CCL4, CCL5, CXCL1, CXCL9, CXCL16, CX3CL1, and XCL1 with LPAR1 were all extracted from the curated databases. The PPI network among LPAR1 and chemokine receptors (Figure 6B) showed that the chemokine receptors, including CCR1, CCR4, CCR5, CXCR2, CXCR3, CXCR6, CX3CR1, and XCR1, had known interactions with LPAR1.

## Discussion

The present study suggests, based on bioinformatics analysis, the importance of LPAR1 in prostate cancer. LPAR1 was lowly expressed in prostate cancer and was significantly related to patient survival. In addition, LPAR1 may be involved in the biological process when immune cells move into the tumor tissues and improve the TME of patients, which impact the development of prostate cancer and the prognosis of patients. Hence, LPAR1 is a potential immune-related biomarker in prostate cancer. Our findings offer deeper insights into the mechanisms of LPAR1 in the development of prostate cancer.

In this study, based on independent datasets in the Oncomine database and TCGA datasets, we examined the LPAR1 expression level in various types of cancer. The differential expression of LPAR1 was seen in a set of cancers between tumor and normal tissues. In the Oncomine database, the results showed that LPAR1 was highly expressed in lymphoma while lowly expressed in prostate, bladder, brain, colon, head and neck, kidney, leukemia, lung, melanoma, and ovarian cancers. The TCGA datasets showed that LPAR1 expression was significantly lowly expressed in PRAD, BLCA, BRCA, COAD, READ, ESCA, HNSC, KICH, KIRC, LIHC, STAD, THCA, and UCEC compared with normal tissues.

The TME has a great impact on the development of cancers (4–6, 31). Studies have shown that the TME, especially the immune microenvironment in tumor, can affect the prognosis of patients (32, 33). However, few reports elaborated the function of LPAR1 in the TME. Through the TIMER database, we found that LPAR1 impacted tumor-infiltrating immune cells in prostate cancer. The LPAR1 expression was significantly negatively related to tumor purity. It may contribute to the infiltration of various immune cells in prostate cancer, including B cells, dendritic cells, NK cells, CD8+ T cells, macrophages, and neutrophils. The MCPcounter and ssGSEA methods revealed that LPAR1 was positively correlated with the infiltration of various types of T cells (γδ T cells, effective memory T Cell, Th1 cells, central memory T cells, and CD8+ T cells), DCs, M1 macrophages, and B cells and negatively correlated with CD56 bright NK cells and Treg cells. The increase of NK cells and CD8+ T cells can help enhance the anti-tumor immunity by secreting various cytokines and releasing perforin and granzyme (34). The infiltration of DCs, the most powerful antigen-presenting cell, can help present antigenic peptides of tumor-associated or tumor-specific antigens to T cells (35). It has been reported that tumor-associated macrophages (36) have a double effect in tumor development. M1 macrophages secrete pro-inflammatory and chemokines, which participate in antigen presentation and immune surveillance, while M2 macrophages secrete inhibitory cytokines. With the LPAR1 upregulated, the M1 macrophages infiltrate into tumor sites and exert an anti-tumor function. The increasing infiltration of B cells helps to eliminate tumor by participating in antibody-dependent cell-mediated cytotoxicity. The CD56 bright NK cells, which are less cytotoxic compared with CD56 dim NK cells, decreased with the upregulation of LPAR1. LPAR1 also downregulates the infiltration of Treg cells, which protect the human body from tumor suppression. Consequently, LPAR1 may play a critical role in regulating TME in prostate cancer by participating in cellular and humoral immunity and motivating the anti-tumor function. Besides that, an analysis of TCGA revealed that the decreased LPAR1 expression was correlated with a poor prognosis in PRAD. A high LPAR1 expression has a correlation with a low HR for poor prognosis, suggesting that LPAR1 is a critical biomarker in prostate cancer.

Concerning biological function, LPAR1 participated in many signaling pathways in tumor cells through GO analysis, for example, passive transmembrane transporter activity, cell motility, metal ion transport, cell differentiation, tube development, G protein-coupled receptor signaling pathway, and chemotaxis. The GSEA results showed that the LPAR1 function was enriched in GO_Leukocyte_Differentiation and GO_Lymphocyte_Mediated_Immunity in prostate cancer. Furthermore, the GSEA on LPAR1 immune-related function indicates that LPAR1 influenced the activation, proliferation, differentiation, and migration of immune cells. It hints that LPAR1 improves the immune response of prostate cancer through various pathways.

LPAR1 has been proven to be associated with chemotaxis, which was also found in the present study. Studies had reported that LPAR1 played a critical role in the LPA-induced chemotactic migration of olfactory ensheathing cells (37). LPAR1-deficient rats showed decreased pulmonary influx of macrophages and neutrophils (38). A previous study showed that LPA promoted microglial migration and induced the secretion of chemokines and pro-inflammatory cytokines, as well as the expression of M1 markers (39, 40). LPA1 and LPA3 receptors play an important role in the synthesis of CXCL1 and its receptor CXCR2 and in the regulation of leukocyte recruitment (14). LPA can induce the chemotaxis of Th1 and Th2 cells (41), and it can promote T cell recruitment through CXCL13 synthesis (42). The chemotaxis of NK cells was also reported to be associated with LPA and LPA receptor (43). We integrated chemokines and chemokine receptors and analyzed the association between LPAR1 and immune cell migration to explore the potential immune-related mechanisms of LPAR1 in prostate cancer. Chemokines control the positioning and the migratory patterns of immune cells. Chemokines are critical for immune cell movement and homeostasis (44). The CX3CL1/CX3CR1 interaction functions in the recruitment of T cell, NK cell, and monocyte. The CX3CL1/CX3CR1 interaction is also associated with the activation of cytotoxic T lymphocytes and NK cell. The CCL4/CCR5 interaction promotes the recruitment of T cell, DC, monocyte, and NK cell, as well as T cell–DC interactions. The CCL22/CCR4 and XCL1/XCR1 interactions are associated with T cell and NK cell recruitment. CCL5/CCR1 takes part in macrophage and NK migration and T cell–DC interactions. The CCL23/CCR1 interaction is about monocyte, neutrophil, and T cell migration. CXCL9/CXCR3 promotes the recruitment of effector T cell. CXCL1/CXCR2 and CXCL16/CXCR6 are associated with neutrophil recruitment and natural killer T cell migration, respectively. In the present study, LPAR1 was not only positively correlated with those chemokine/chemokine receptors but also had known or predicted interactions with them based on the PPI network, indicating that LPAR1 may increase the immune infiltrates of tumor through regulating the migration of immune cells in prostate cancer.

As far as we know, this is the first study to elaborate the potential functions of LPAR1 and its association with tumor-infiltrating immune cells by using integrated bioinformatics analysis. However, this present study had limitations. Further molecular experiments are deserved to verify the mechanisms of LPAR1 and its effects on the clinical outcome in prostate cancer. Moreover, it is also important to integrate and elaborate the association between LPAR1 and chemokines/chemokine receptors, which can help us better understand the TME, especially the immune microenvironment in tumors.

## Data Availability Statement

All datasets were extracted from The Cancer Genome Atlas (https://tcga-data.nci.nih.gov/tcga/) and Oncomine (https://www.oncomine.org/) websites, so the ethical statement was already admitted and further evaluation of it was not necessary.

## Author Contributions

KY and JS conceived and designed the project. JS, DJ, SY, XZ, JW, YLi, YS, and YLu analyzed the data. JS wrote the manuscript with input from all the other authors.

## Conflict of Interest

The authors declare that the research was conducted in the absence of any commercial or financial relationships that could be construed as a potential conflict of interest.
