# Supplementary figures and images for: LPAR1, Correlated With Immune Infiltrates, Is a Potential Prognostic Biomarker in Prostate Cancer
Source: Front Oncol. 2020 Jun 10;10:846. doi: 10.3389/fonc.2020.00846 (PMC7325998; doi:10.3389/fonc.2020.00846)

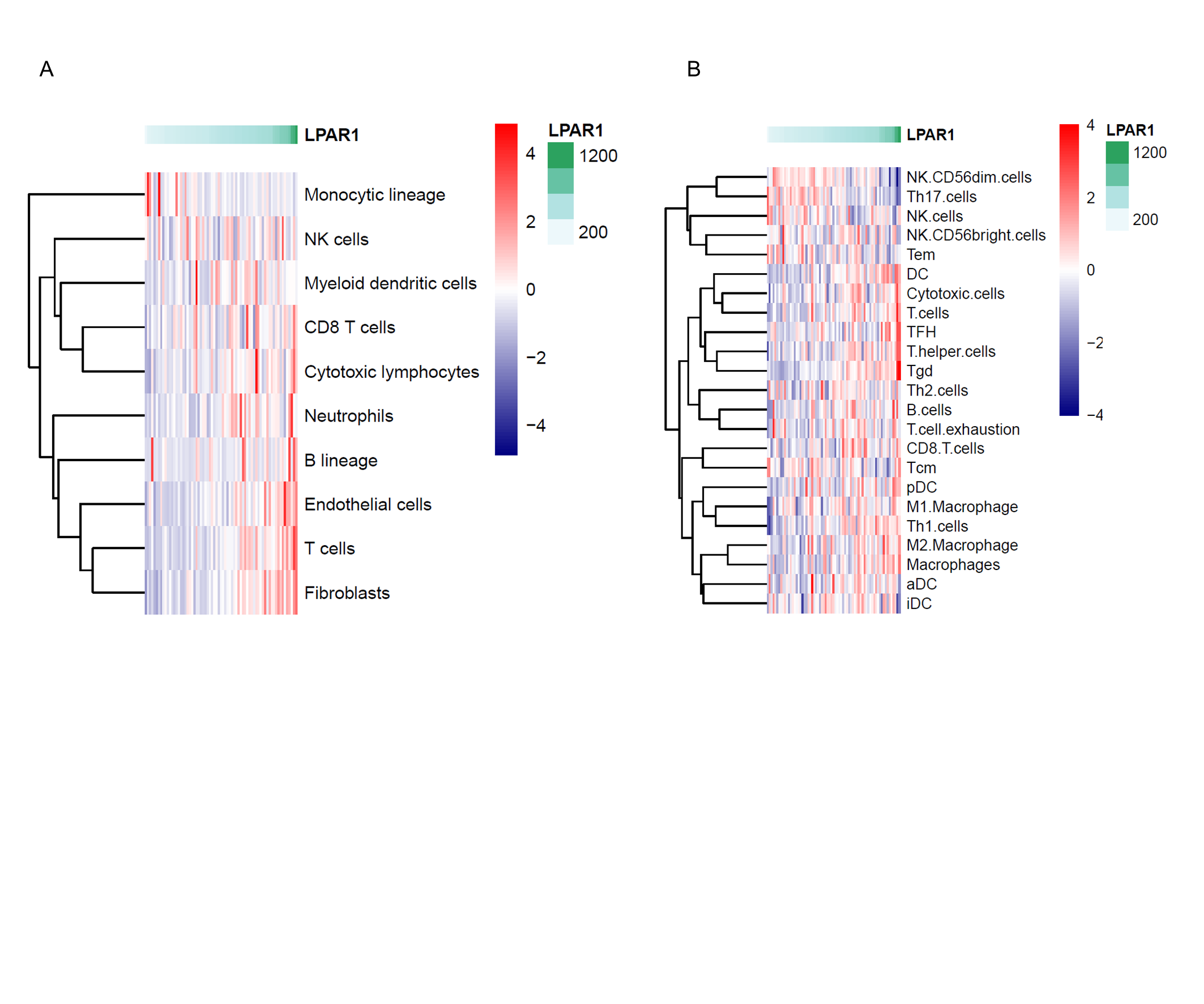

Supplement: Supplementary file 3 [file Image_2.TIF]

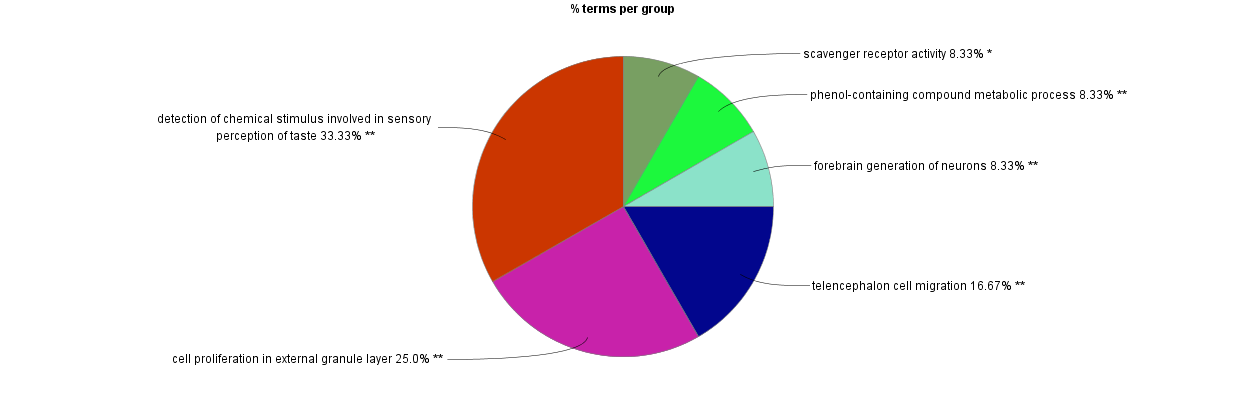

Supplement: Supplementary file 4 [file Image_3.PNG]
